# Effective litmus gene test for monitoring the quality of blood samples: Application to Alzheimer’s disease diagnostics

**DOI:** 10.1038/s41598-017-17293-2

**Published:** 2017-12-04

**Authors:** Sung-Mi Shim, Jong-Hoon Kim, Jae-Pil Jeon

**Affiliations:** 1 Division of Brain Diseases, Center for Biomedical Sciences, Korea National Institute of Health, 187 Osongsaengmyeong 2-ro, Osong-eup, Heungdeok-gu, Cheongju-si, Chungcheongbuk-do, 28159 Republic of Korea; 2 Department of Biotechnology, College of Life Sciences and Biotechnology, Korea University, 145 Anam-ro, Seongbuk-gu, Seoul, 02841 Republic of Korea

## Abstract

Gene expression profiles reflect the biologically diverse activities of cells under specific cell environments. Using the transcriptional response of cultured cells to blood composition, we developed a litmus gene assay to discriminate blood samples reflecting different sample qualities or disease conditions. This cell-based litmus gene assay identified six genes (*CCL20*, *CEMIP*, *IL1B*, *IL8*, *PRG2*, *PTGS2*) as potential biomarkers of plasma quality control and the *SPC25* gene as a diagnostic biomarker of Alzheimer’s disease (AD). In addition, the *SPC25* gene expression level was significantly increased in the cell-based assay using serum samples from patients with mild cognitive impairment (MCI). In conclusion, we demonstrated the effectiveness and potential of a litmus gene assay to detect the orchestrated effects of circulating systemic factors, leading to the successful diagnosis of AD and MCI. This method is broadly applicable to the diagnosis of disease subtypes or patho-physiological stages of complex diseases and tumors.

## Introduction

Peripheral blood contains a variety of systemically-acting factors that are in balance in a healthy human body. Disease development and progression are often accompanied by changes in the composition of these peripheral systemic factors, including cytokines, antibodies, cell-free nucleic acids, platelets, and extracellular vesicles such as microvesicles and exosomes. These systemic factors are known to play roles in regulating carcinoma progression or ageing^1–3^.

Currently, most blood-based diagnostic methods rely on tests to measure directly the blood concentrations of specific factors associated with diseases^4–6^. The principle behind these direct methods is suited to the discovery of a single factor or a limited set of individual factors related to disease conditions. However, this methodology may not ensure the discovery of disease-specific biomarkers reflecting the orchestrated effects of multiple systemic factors as well as local target tissues on the pathophysiological conditions associated with the disease. A few attempts have been made to identify the blood-based signatures of direct or indirect plasma-induced transcription in diabetes^7^, which is the most common chronic complex disease. Thus, to develop more effective diagnostics for complex diseases, including age-related neurodegenerative diseases, an alternative diagnostic approach applicable to the discovery of harmonized biomarkers reflecting systemic disease conditions may be needed.

Cultured cells are capable of specifically sensing diverse cell environments at the cellular and molecular levels. Specifically, the transcriptional response of the cell to environmental stimuli is highly dynamic and rapid. Gene expression signatures have become a powerful tool to dissect disease subtypes or disease progression stages in clinical settings^8–10^. Thus, specific gene expression signatures have provided potential diagnostic biomarkers of complex diseases and tumors. These approaches have largely focused on the transcriptomic profiles of specific disease-affected tissues or cells, such as hepatocytes for liver cancer^11,12^, lymphocytes for Burkitt’s lymphoma^13,14^, pancreatic beta islets for diabetes^15^, or hippocampal neurons for Alzheimer’s disease (AD)^16,17^. However, disease conditions may not only affect disease-specific local tissues but also disturb the balanced physiological system. For example, AD is known to be a multifactorial neurodegenerative disease that affects both the central nervous system and the periphery^18^. Therefore, most efforts have been focused on identifying blood-based biomarkers for AD. However, until now, effective blood-based AD biomarkers have not been successfully applied in clinical practice.

## Results

### Identification of plasma quality control biomarkers

To develop a new blood-based diagnostic method, we analyzed the changes in the transcriptional responses of genes in cultured cells treated with blood fractions of different qualities (Fig. S1-1). To do this, decayed plasma test samples that were exposed to room temperature for an undetermined number of days were employed for a microarray analysis of plasma-treated cell cultures to select differentially expressed genes (DEGs) relative to levels in cells treated with fresh cryopreserved plasma control samples (−80 °C). Here, we tested whether some of the DEGs could be used as quality control biomarkers of plasma samples.

**Figure 1:**
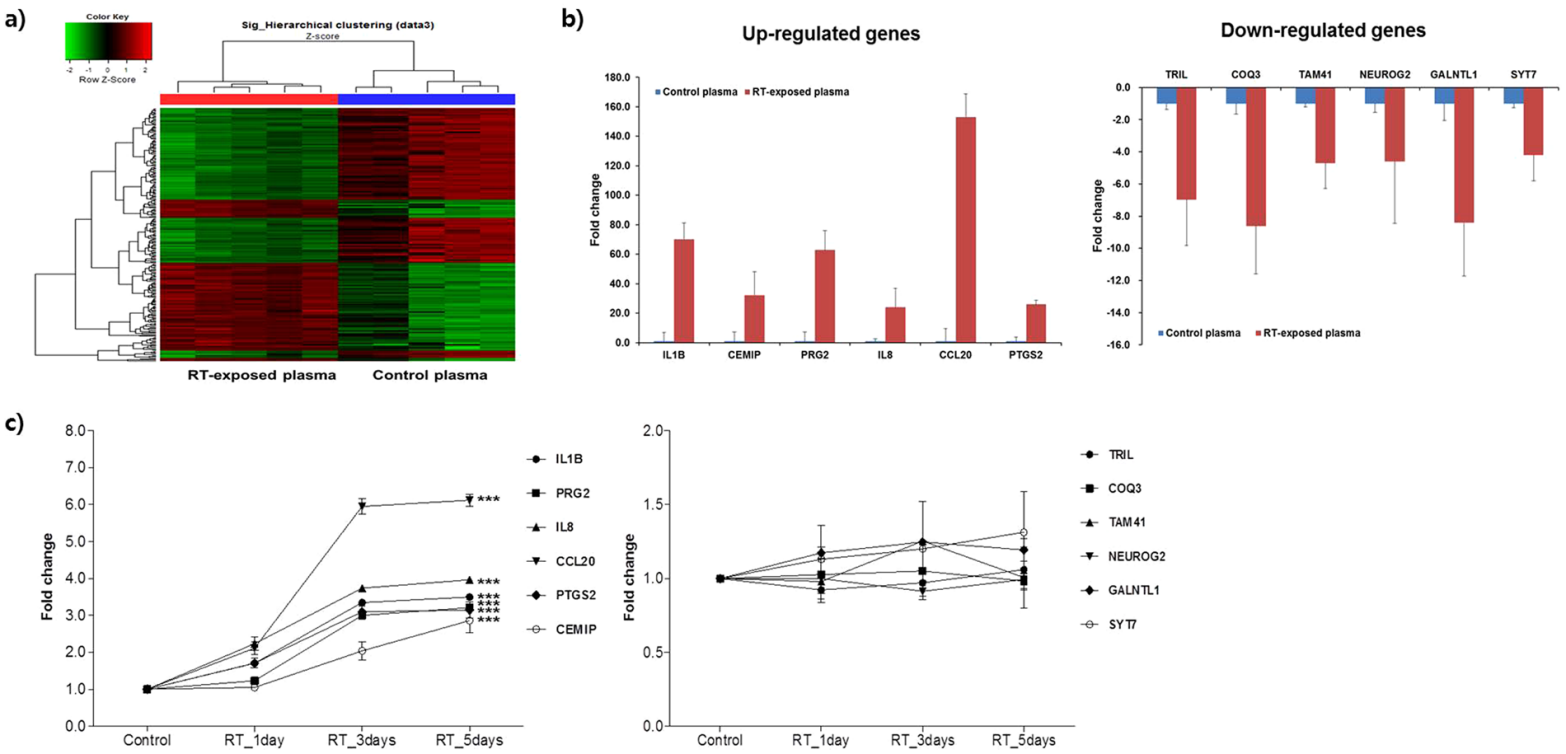
Effectiveness of the litmus gene assay for plasma quality control. (**a**) Hierarchical clustering of gene expression changes in decayed (exposed to room temperature for an unknown number of days) or cryopreserved plasma samples. (**b**) PCR validation of the microarray data. Six up-regulated and six down-regulated differentially expressed genes (DEGs) were validated by real-time PCR in neuronal cells treated with decayed or cryopreserved plasma samples. The data are expressed as the mean ± SD from three replications. (**c**) Gene expression changes in response to the length of exposure time at room temperature (RT). Selected genes were up- or down-regulated in neuronal cells treated with plasma samples stored for different periods of time (0, 1, 3, 5 days at RT). The fold change was calculated relative to the control sample (0 day at RT). The data are expressed as the mean ± SD from three replications. The significant differences in the expression of genes at all points of were analyzed using ANOVA. ***p < 0.0001.

In the present study, human neuroblastoma cells (SH-SY5Y) were treated with decayed plasma samples under standard cell culture conditions (37 °C, 5% CO_2_) for 1 day. We used a modified culture medium that was supplemented with 10% human plasma rather than 10% fetal bovine serum (FBS). Our microarray analysis of human plasma-treated cells for the test plasma (n = 5, decayed) and control plasma (n = 5, cryopreserved) identified a total of 207 DEGs (p < 0.05, ≥2-fold change) (Fig. 1a). The microarray results were then validated by qPCR using a selected set of 12 DEGs (Fig. 1b). Next, the accelerated decay tests were performed for fresh cryopreserved plasma samples (n = 5), which were exposed to room temperature for different times (0, 1, 3, and 5 days). Only six up-regulated DEGs (*CCL20*, *CEMIP*, *IL1B*, *IL8*, *PRG2*, *PTGS2*) were confirmed to exhibit significant changes in gene expression in cell culture in response to the decayed plasma samples (Fig. 1c). These changes in gene expression were dependent on the exposure time of the frozen plasma samples to room temperature. These results demonstrate that the transcriptional response of human plasma-treated culture cells could be basis of a new gene-based assay using a sensor cell to monitor the quality of plasma samples. Here, we named this new gene-based assay the “QC Litmus Gene Test” for plasma quality control (QC). As in the traditional litmus paper test to determine if a solution is acidic or basic, this litmus gene test can be applied to test whether blood samples were stored under different conditions

### Application of the litmus gene assay to AD diagnosis

**Figure 2:**
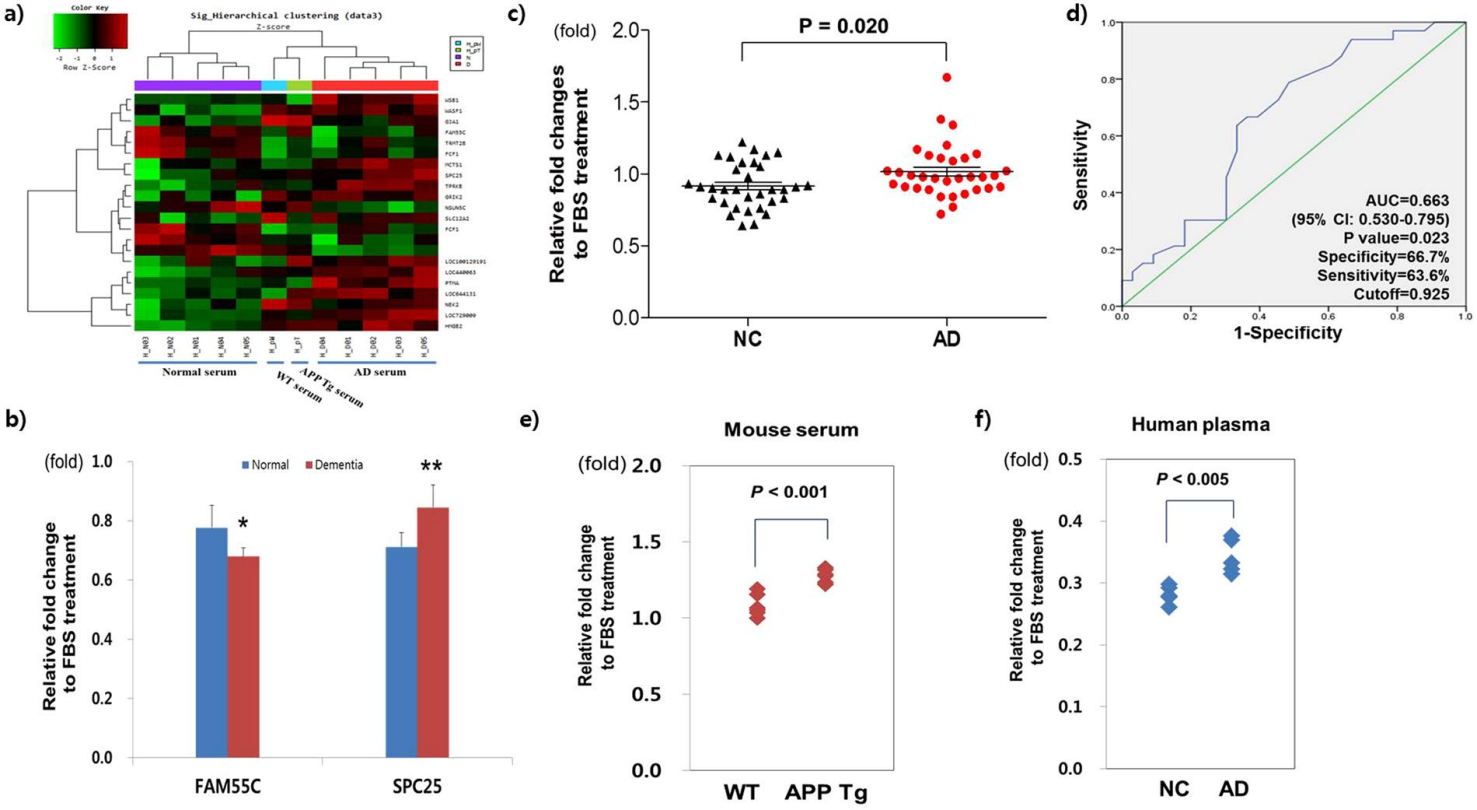
Applications of the litmus gene assay to AD diagnostics. (**a**,**b**) Discovery and validation of the AD litmus gene. (**a**) Hierarchical clustering of gene expression in neuronal cells treated with AD serum samples from cognitively normal subjects (Normal), AD patients (AD), and pooled mouse serum samples from wild-type (WT, n = 5) and APPdE1PS1 transgenic mice (APP Tg, n = 5). (**b**) Validation of candidate AD litmus genes. Gene expression in human serum-treated cells was normalized to that of FBS-treated neuronal cells (SH-SY5Y). qPCR was performed for the identical sample sets of human sera (Normal, n = 5; AD, n = 5) used in the microarray experiment. *p < 0.05, **p < 0.001. (**c**,**d**) Diagnostic performance of the AD litmus gene (*SPC25*) for AD. AD sera (n = 33) induced a significantly higher activity of *SPC25* gene expression in neuronal cells compared to that induced by normal sera (n = 33). Normal and AD serum samples were matched for age and sex. P-values indicate significance following Student’s t-test (**c**). AD subjects were distinguished from normal subjects, with an AUC of 0.663 (p = 2.32E-02, 95% CI: 0.530~0.795) in the ROC analysis. (**d**–**f**) Extended application of the litmus gene assay to serum samples from dementia mouse models and plasma samples from AD patients. The AD Litmus Gene Assay results showed that sera from the AD mouse model (APP Tg, APPswePS1dE9 transgenic) significantly increased *Spc*25 gene expression in mouse neuronal cells (T4 cells) compared to that induced by sera from wild-type mice (WT) (**e**). In addition, AD plasma samples exhibited significantly higher gene expression of *SPC*25 in plasma-treated neuroblastoma cells (SH-SY5Y) (**f**). The *GAPDH* gene was used as an internal load control in real-time qPCR. *GAPDH*-normalized gene expression levels in mouse serum- and human plasma-treated cells were used to calculate the relative gene expression levels of *SPC*25 in reference to gene expression in FBS-treated cells. qPCR was performed in duplicate wells with the identical set to human subjects used in microarray experiment. P values indicate significance following t-tests.

To demonstrate the effectiveness of this litmus gene assay for classifying blood samples of different qualities, we tested disease serum samples for the diagnosis of AD. First, microarray analysis was performed using neuroblastoma cells treated with human serum samples of the discovery sample set, which included age- (±2) and sex-matched serum samples of AD cases (n = 5) and normal non-dementia controls (n = 5). In total, 27 candidate transcriptional target genes (cutoffs ≥1.2-fold change, p < 0.05) were found to be differentially expressed in response to the AD serum treatment (Fig. 2a). Out of these DEGs, the *SPC25* (spindle pole body component 25 homolog) and *FAM55C* (family with sequence similarity 55, member C) genes were confirmed in the same discovery sample set (Fig. 2b).

When expanded to the validation sample sets (33 normal subjects and 33 AD patients) from the community-based Ansan Geriatric Study^19^, the AD Litmus Gene Assay showed that the gene expression of *SPC*25 was significantly up-regulated (p = 0.020) in AD serum-treated cells (Fig. 2c), but not *FAM55C*. According to our ROC analysis, the AD Litmus Gene Assay did not show a strong power of discrimination between AD serum and normal samples, with an area under the curve of 0.663 (95% CI: 0.530–0.795, p = 0.023) (Fig. 2d). In the AD Litmus Gene Assay, the transcriptional response of the target gene (*SPC*25) to human sera was normalized by the expression level of the target gene in 10% FBS-treated cells. This FBS-normalized gene expression allowed for the control of any batch effect of cell culture conditions. Thus, FBS-normalized *SPC*25 gene expression can be used in different laboratories for AD diagnostics as a relatively robust indicator of the pathogenic potential of AD.

Next, we also confirmed the significantly higher expression of the AD litmus gene (*Spc*25) in mouse neuronal T4 cells treated with mouse sera from 16-month-old APP transgenic mice, an Alzheimer’s disease model (APPswe/PS1dE9) (Fig. 2e). When the AD Litmus Gene Assay applied to plasma samples rather than serum samples, the *SPC*25 gene expression level was also significantly increased in the AD plasma-treated cells (Fig. 2f). This result suggests that plasma samples are also suitable for the AD Litmus Gene Assay.

### Effectiveness of the AD Litmus Gene Assay for MCI diagnostics

**Figure 3:**
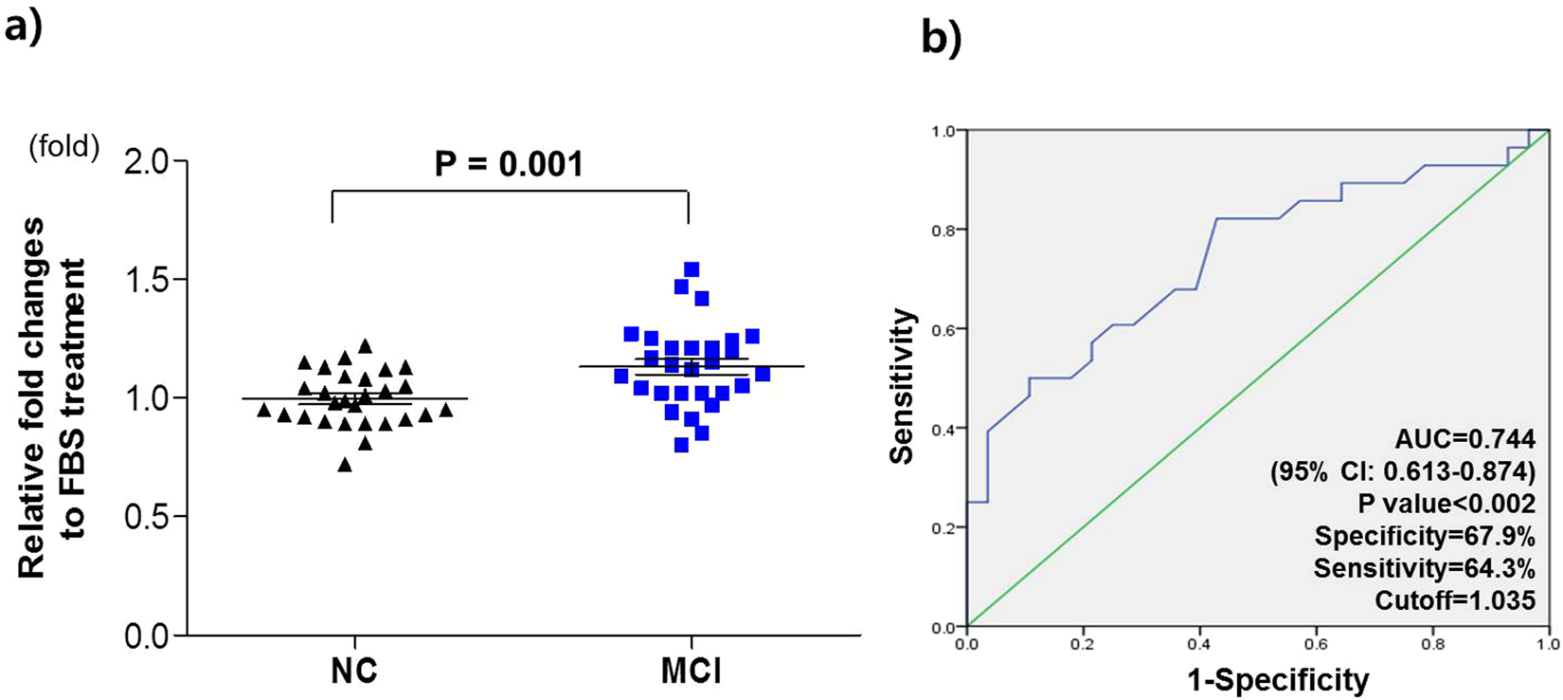
Diagnostic performance of the AD litmus gene (*SPC25*) for MCI. (**a**) MCI sera (n = 28) induced a significantly higher activity of *SPC25* gene expression in neuronal cells compared to that induced by normal sera (n = 28). Normal and MCI serum samples were matched for age and sex. P-values indicate significance following Student’s t-tests. (**b**) MCI subjects were distinguished from normal subjects with an AUC of 0.744 (p = 1.75E-03, 95% CI: 0.613~0.874) in the ROC analysis.

To further prove the diagnostic performance of the AD Litmus Gene Assay, we tested its applicability to the diagnosis of mild cognitive impairment (MCI), a prodromal phase of Alzheimer’s disease. The AD Litmus Gene Assay showed that MCI serum-treated neuronal cells exhibited a significantly higher gene expression of *SPC25* than normal serum-treated neuronal cells (p = 0.001) (Fig. 3a). The ROC curve analysis showed that this AD Litmus Gene Assay can differentiate MCI serum samples from normal samples, with an area under the curve of 0.744 (95% CI: 0.613–0.874, p < 0.002) (Fig. 3b). Taken together with results from AD serum treatment, this result supports that *SPC*25 gene expression is a potential target of the AD Litmus Gene Assay to discriminate AD serum samples from normal control serum samples.

## Discussion

In the present study, we developed a cell-based litmus gene assay, which can be used for biomarker discovery. This assay exploited the gene expression levels of a particular sensor cell to monitor the molecular effects of target biofluids, including serum and plasma samples. Here, we demonstrated the effectiveness and potential of this litmus gene assay for biomarkers of plasma quality control as well as AD and MCI diagnostics. This litmus gene assay involves two steps: treating cultured cells with the target blood samples and subsequently measuring the gene expression levels of particular genes in the cells, evaluating indirectly the harmonized effects of the target biofluid samples originating from patients or reflecting different sample quality levels. In this regard, this assay might be inconvenient compared to conventional methods that measure directly biomarker concentrations in blood samples. However, considering that there is no gold standard biomarker or tool available for plasma quality control or blood-based AD diagnostics, this indirect method can provide an alternative way to identify disease biomarkers.

Many pre-analytical variables such as storage time and temperature can affect the experimental results of stored plasma or serum samples^20–24^. This issue is particularly important when searching for blood-based biomarkers of diseases. For examples, serum vascular growth factor (VEGF) has been suggested as a blood-based biomarker of various diseases, including Alzheimer’s disease^25^, colorectal cancer^26^, and depression^27^. However, the serum VEGF level is very unstable, depending on storage conditions such as the freeze-thaw cycle^28^. Therefore, studies aimed at discovering blood-derived biomarkers require high-quality blood samples in order to develop reliable biomarkers. We were also able to confirm the quality of blood samples used in the AD Litmus Gene Assay using the QC Litmus Gene Assay in the same cell line, SH-SY5Y. Indeed, the expression levels of the six litmus QC genes were not different between the two groups of AD cases and cognitively normal controls (data not shown).

A few reports have described the use of the direct transcriptional profile in whole blood^21^ or peripheral blood mononuclear cells (PBMCs)^29^ for the diagnosis of AD. In these studies, a microarray test of 96 differentially expressed genes was employed for the early diagnosis of AD and exhibited an accuracy of 71.6%^30^ even with the use of a gene panel including 96 DEGs. The direct transcriptional responses of PBMCs can be sensitive to the health conditions of donors because PBMCs are mostly composed of immune cells, including T-cells and B-cells. Therefore, our indirect method using well-controlled cell culture conditions may be more reliable than the direct blood-based test measuring gene expression patterns in PBMCs.

The *SPC25* gene encodes a component of the NDC80 kinetochore complex that may be involved in kinetochore-microtubule interactions and spindle checkpoint activity^31^. Although there was a report that *SPC25* was upregulated in aged monocytes from rats^32^, it is not known if *SPC25* is associated with neurodegenerative diseases such as AD. The involvement of *SPC25* in AD pathogenesis remains to be determined. Moreover, the molecular pathways upstream of *SPC25* gene expression may provide novel insights into AD pathogenesis. This suggests that the transcriptional regulatory machinery of the *SPC*25 gene is a target of serum factors from AD patients.

In general, conventional blood-based diagnostic approaches have focused on directly measuring specific blood factors as target molecules for diagnostic assays. In contrast, we developed an alternative approach to AD diagnostics that relies on detecting the orchestrated effects of circulating systemic factors derived from disease-specific conditions. By analogy, this indirect detection method was named the litmus gene assay after the traditional litmus paper test for acidity. Here, we demonstrated that the principle of the litmus gene assay was applicable to diagnostic biomarkers for plasma quality control and AD pathogenesis. For AD diagnosis, this novel AD Litmus Gene Assay needs to be improved to optimize detection sensitivity and accuracy in cell culture systems. For example, in addition to the replication experiment using different AD cohort samples, this AD Litmus Gene Assay can be also further improved by using reporter vector systems containing a promoter modification of the *SPC25* gene and by selecting the best sensor cell type that responds to circulating systemic factors from AD patients.

Our study had several limitations. First, we used the SH-SY5Y neuroblastoma cell line to identify the plasma QC litmus genes. The reason that this neuronal cell line was used was that the final goal of this study was to apply this method to diagnosis of brain diseases such as AD. However, there may be better cell lines derived from fibroblasts or lymphocytes for checking the quality of plasma or serum samples. Further studies will be needed to improve the efficacy of this assay in different cell types. Second, we initially developed the concept of this litmus gene assay using a relatively small size of samples. In future studies, a larger sample size and different cohort samples may help find more candidate AD litmus genes and improve diagnostic accuracy. Third, the QC Litmus Gene Assay needs to be tested using various sample storage conditions (e.g., storage temperature, long-term storage, freeze-thaw cycles) in order to build a predictive model for sample quality.

The concept of the litmus gene approach can be extended to many research areas. For example, the target genes of different litmus gene approaches would provide clues for novel diagnostic and therapeutic targets for different diseases^33^. Some cancer studies reported that sera from cancer patients and healthy individuals induced different responses in immune-related gene expression or protein release in mitogen-induced peripheral blood mononuclear cells (PBMCs)^34,35^. For example, they showed that sera from lung cancer patients down-regulated interleukin-2 receptor subunit alpha (IL-2Rα) gene expression and inhibited interleukin-1 beta (IL-1B) and IL-2 release in concanavalin A-stimulated PBMCs from healthy donors compared to the sera from normal controls^35^. Therefore, as in the litmus gene assay, the harmonized effect of serum factors from cancer patients could be utilized to develop diagnostic assays for cancer biomarker discovery.

In conclusion, we developed a litmus gene assay by exploiting the specific transcriptional responses of cultured cells treated with blood fractions and demonstrated the effectiveness of this assay in applications of plasma quality control and AD diagnosis. Such transcriptional responses depended on the serum factors in blood samples. The principle of the litmus gene assay was based on the specific gene expression of cultured cells in response to the biochemistry of peripheral blood samples of different qualities (e.g., from different storage conditions and different disease types and stages). The fundamental merit of this litmus gene assay is that when applied to clinical settings, it is not necessary to know the specific compositions of each peripheral blood fraction. Therefore, this assay can be principally applied to any type of disease, such as complex diseases, cancer, and even infectious diseases, as well as any type of liquid biopsy sample (e.g., blood fractions, cerebrospinal fluid, and saliva) or conditioned media from stem cell culture for therapy. Thus, this method is broadly applicable to diagnostics in clinical settings and quality management systems of biospecimens and stem cell banks.

## Methods

### Development of a litmus gene assay for plasma quality control

To identify transcriptional target genes of the cultured cells in response to various serum factors in blood, we analyzed the gene expression profiles of human neuroblastoma cells that were cultured in the DMEM plus 10% human plasma samples, either the test decayed samples (n = 5) or fresh cryopreserved control samples (n = 5) (Fig. S1-1). The plasma samples used in this experiment were obtained from study subjects in the community-based Ansan Geriatric Cohort Study^19^. Our microarray analysis identified 207 differentially expressed genes (DEGs) with cutoffs of p < 0.05 and over 2-fold changes (Fig. S1-2, Table S1-1). Functional analysis showed that these DEGs were enriched for the functions vascular development, angiogenesis, and cell death (Table S1-2). In this study, we developed a new cell-based assay system and target genes to assess the sample quality of stored plasma samples. The new assay system and biomarkers can be utilized as a practical tool to monitor the quality of cryopreserved plasma samples.

#### Blood samples

The blood samples used in this study were obtained from the dementia cohort study or the population-based geriatric cohort study^19^. For microarray experiments, the discovery sample set included the test plasma samples (n = 5) that were exposed to room temperature for an unknown number of days and the control plasma samples (n = 5) that were cryopreserved at the typical ultra-low temperature (−80 °C) for long-term storage.

#### Cell culture for the plasma QC Litmus Gene Assay

The human neuroblastoma cell line (SH-SY5Y) was maintained in DMEM (Dulbecco’s modified Eagle’s medium) supplemented with 10% FBS (Gibco, Grand Island, NY, USA), unless otherwise stated, and antibiotics (100 U/ml penicillin, 100 µg/ml streptomycin) at 37 °C with 5% CO_2_. For the litmus gene assay, cells were trypsinized with TrypLE Express (Gibco) and washed with fresh DMEM twice. Cells were seeded in DMEM plus 10% human plasma at a density of 2 × 10^6^ cells/ml in a 6-well plate and then incubated at 37 °C with 5% CO_2_ for 24 hours.

#### Microarray and qPCR for the plasma QC Litmus Gene Assay

Total RNA was extracted from the human plasma-treated SH-SY5Y cells with an RNeasy plus kit (Qiagen, Hilden, Germany). The Illumina Human HT-12 v4.0 BeadChip was used for the microarray experiment and analysis according to the manufacturer’s instructions (Illumina Infinium). The chip signals were scanned in the Illumina Image BeadArray Reader using Illumina BeadStudio. After quantile normalization of signal intensities, DEGs were selected with cutoffs of p < 0.05 and >2-fold changes. Next, the gene expression of selected DEGs (n = 12) was validated by qPCR in the same plasma samples that were used in the microarray experiment (Table S1-3). For qPCR validation of the microarray data, 4 µg of total RNA was used to synthesize cDNA using cDNA EcoDry^TM^ Premix (Oligo dT) (Clontech, Mountain View, CA, USA), which was then used for quantitative real-time PCR reactions (qPCR). qPCR was performed in a 20 µl reaction mixture containing 10 µl of 2X SYBR Green mixture (Applied Biosystems, Foster City, CA, USA) and 1 µM appropriate primers (Table S1-3). qPCR reactions included a cycle of 95 °C for 10 min, 40 cycles of 95 °C for 15 sec and 60 °C for 1 min, and a cycle of 95 °C for 10 min. Expression levels of particular genes were normalized by a reference gene (human or mouse GAPDH gene) as a load control in the qPCR reaction. After obtaining the differences in the GAPDH-normalized Ct values between the test and control groups, the transcriptional response of the particular gene was represented as a target gene expression level of human plasma or serum-treated cells in reference to that of FBS-treated cells. When the overall gene expression levels of the plasma QC genes were analyzed, the average fold changes in gene expression of six upregulated genes (*IL-1B*, *PRG2*, *IL-8*, *CCL20*, *PTGS2*, *CEMIP*) increased during the time period of exposure of plasma samples to room temperature (Fig. S1-3).

### Development of a gene-based assay for Alzheimer’s disease diagnostics

To demonstrate the effectiveness and potential of the litmus gene assay to detect the orchestrated effects of circulating systemic factors, we tested whether this assay can be applied to the successful diagnosis of Alzheimer’s dementia. Basically, the same principle as the plasma quality control was used to discriminate MCI or AD blood samples from normal control blood samples (Fig. S2).

#### Study subjects

This study included elderly Korean participants recruited for a general population-based geriatric cohort study. Its study design, sampling, concept, and consent were described elsewhere^12^. At the initial recruitment phase starting in 2002, participants (n = 2,767) were enrolled for general interviews and completed only the Korean Mini-Mental State Examination (K-MMSE) as a neuropsychological test. Subsequent follow-up studies were conducted four times approximately every 2~3 years from 2003 to 2010. Here, the present study used study subjects who were over 60 years of age and participated in the follow-up study from 2009 to 2010. For microarray experiments, the discovery sample set included Alzheimer’s disease (AD) cases (n = 5) and cognitively normal control (n = 5) groups, which were matched by sex and age (+/−2), did not have diabetes and hypertension, and did not take any medicine on the date of survey. For further validation experiments, the AD validation sample set included the AD (n = 32) and cognitively normal (n = 32) subjects, and the MCI validation sample set included the MCI (n = 28) and cognitively normal (n = 28) subjects. These validation sample sets were matched for age (+/−2) and sex (Table S2-1). Diagnosis of dementia was based on the guidelines of the Diagnosis and Statistical Manual of Mental Disorders, fourth edition (DSM-IV). Diagnosis of MCI was based on the criteria of Petersen^36,37^. According to the conventional Petersen/Winblad criteria, there are several different subtypes of MCI, including the two major categories of amnestic (characterized by primary memory impairment) and non-amnestic (non-memory impairment). Thus, the present study used blood samples from study subjects with amnestic MCI. The criteria for amnestic MCI are as follows: memory complaints, objective memory impairment on a delayed recall test, relatively normal general cognitive function, normal or only minimally impaired activities of daily living, and not demented. The institutional review board of Korea Centers for Disease Control and Prevention (KCDC) approved the research protocol, and written informed consent was obtained from all subjects after the nature of the study and its procedures had been explained.

#### Animals

Double-transgenic amyloid precursor protein (APP) Swedish/presenilin (PS)-1ΔE9 transgenic (Tg) mice were purchased from the Jackson Laboratory (Bar Harbor, ME, USA) and maintained by crossing with wild-type (WT) mice with a C57BL/6 J background. APP transgenic mice and WT mice at 16 months of age were used in the present study, and blood samples were collected from their tail vein. All studies were conducted with a protocol approved by the local Institutional Animal Care Use Committee in compliance with Korea National Institute of Health guidelines for the care and use of experimental animals.

#### Cell culture for the AD Litmus Gene Assay

The human neuroblastoma cell line (SH-SY5Y) was maintained in DMEM (Dulbecco’s modified Eagle’s medium) supplemented with 10% FBS (Gibco, Grand Island, NY, USA), unless otherwise stated, and antibiotics (100 U/ml penicillin, 100 µg/ml streptomycin) at 37 °C with 5% CO_2_. Mouse neuronal T4 cells were maintained in DMEM plus 10% FBS at 33 °C with 5% CO_2_. For the treatment of SH-SY5Y cells with human plasma or serum, cells were washed with PBS twice and trypsinized with TrypLE Express (Gibco). After adding DMEM plus 10% FBS for trypsin inactivation, trypsinized cells were washed twice with fresh DMEM. Culture media were prepared to contain DMEM plus 10% human plasma or serum supplemented with antibiotics. For the treatment of SH-SY5Y cells with serum from WT or APP Tg mice, the serum samples were pooled from 5 WT mice or 5 APP Tg mice at 16 months of age. The culture media were supplemented with 10% mouse serum instead of 10% FBS. The pooled WT and APP Tg sera were used as a reference sample set for microarray analysis. SH-SY5Y cells were seeded at a density of 2 × 10^6^ cells/ml per well in a 6-well plate and then incubated at 37 °C with 5% CO_2_ for 24 hours. For the treatment of T4 cells with serum from mice, 5 × 10^5^ cells per well were seeded at a density of 1 × 10^6^ cells/ml in a 12-well plate and incubated at 33 °C with 5% CO_2_ for 24 hours. To control for batch effects due to culture conditions, the treatment and subsequent qPCR assays included reference cells treated with 10% FBS.

#### Microarray and qPCR for the Litmus AD gene assay

Microarray and qPCR experiments were conducted as described in the previous plasma QC experiments, except with more stringent cutoffs (p < 0.05 and fold changes ≥ |1.2|) for selecting DEGs from the microarray chip signals (Table S2-2). Next, the gene expression of selected DEGs was validated by qPCR using appropriate primers (Table S2-3) in the cohort samples. Out of the DEGs tested in the chip validation, two genes (*SPC25* and *FAM55C*) were validated in the subsequent qPCR using the same serum samples as used in the microarray experiment.

### Statistical analysis

All statistical analyses were performed using the Statistical Package for Social Science (version 12.0; SPSS Inc, Chicago, USA). Significant differences between groups were assessed by Student’s t-test or ANOVA. Linear regression analysis was performed after adjusting for age, sex, and education to evaluate the association between *SPC25* gene expression level and cognitive function. Receiver operating characteristic curve (ROC) analysis was conducted to evaluate the diagnostic accuracy of the litmus gene assay. Statistically significance was considered at p < 0.05.

## Electronic supplementary material

Supplementary figures and tables
