# Supplementary material for: Effective litmus gene test for monitoring the quality of blood samples: Application to Alzheimer’s disease diagnostics
Source: Sci Rep. 2017 Dec 4;7:16848. doi: 10.1038/s41598-017-17293-2 (PMC5715155; doi:10.1038/s41598-017-17293-2)
Supplement: Supplementary file 1 — Supplementary figures and tables [file 41598_2017_17293_MOESM1_ESM.pdf]

Effective litmus gene test for monitoring the quality of blood samples: Application to Alzheimer's disease diagnostics

Sung-Mi Shim[1,2], Jong-Hoon Kim[2], Jae-Pil Jeon[1,*]

[1] Division of Brain Diseases, Center for Biomedical Sciences, Korea National Institute of Health, Osong, Republic of Korea

[2] Department of Biotechnology, College of Life Sciences and Biotechnology, Korea University, Republic of Korea

[*]Correspondence to Jae-Pil Jeon

Address: 187 Osongsaengmyeong-2-ro, Osong-eup, Heungdeok-gu, Cheongju-si, Chungcheongbuk-do, Republic of Korea 363-951

Email: jaepiljeon@hanmail.net, Tel: 82-43-719-8632, Fax: 82-43-719-8602

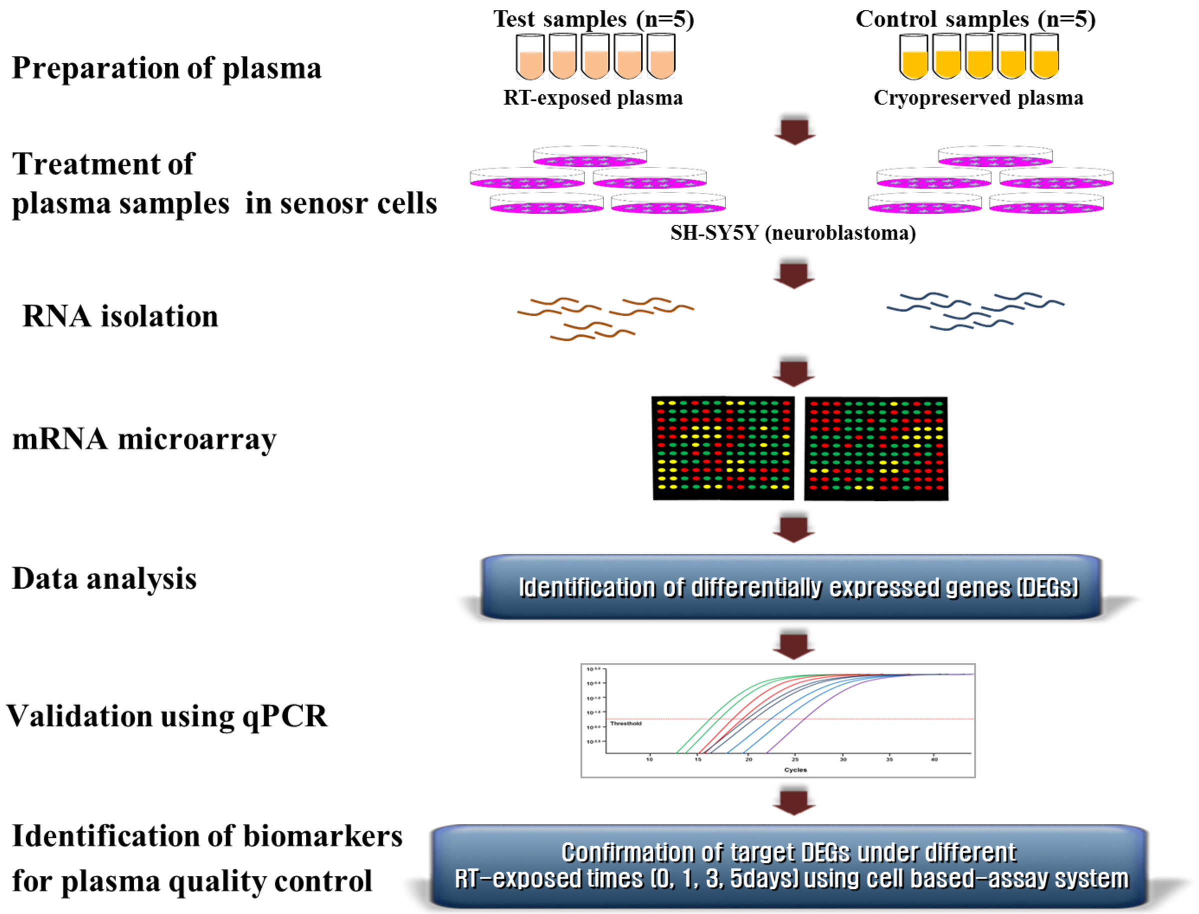

Figure S1-1. Experimental design using a cell-based assay system for plasma quality control

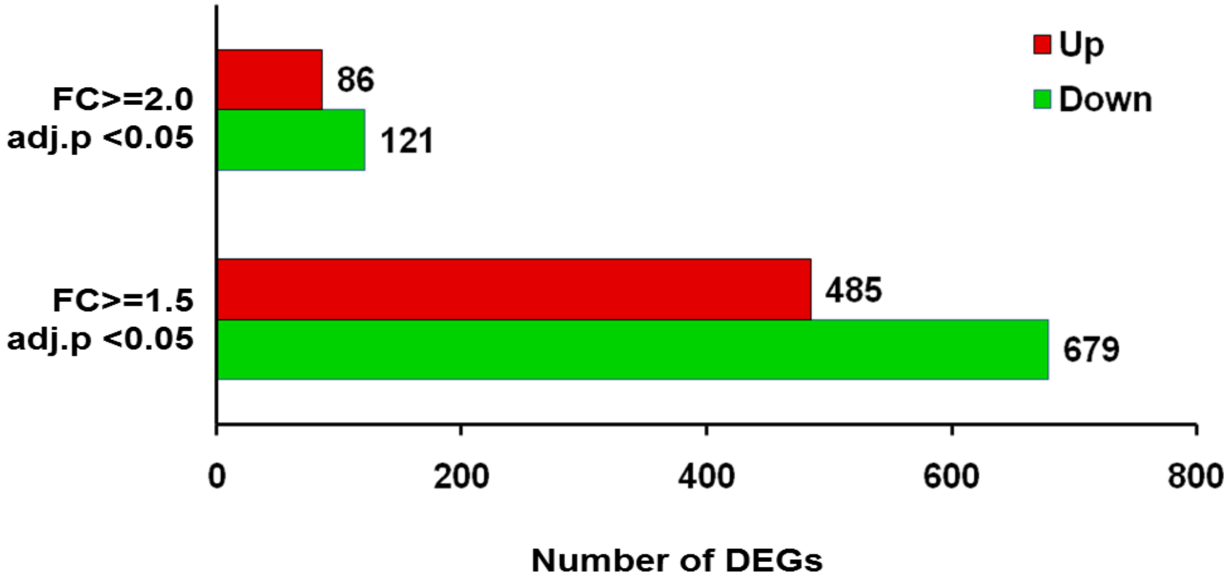

Figure S1-2. Summary of differentially expressed genes (DEGs)

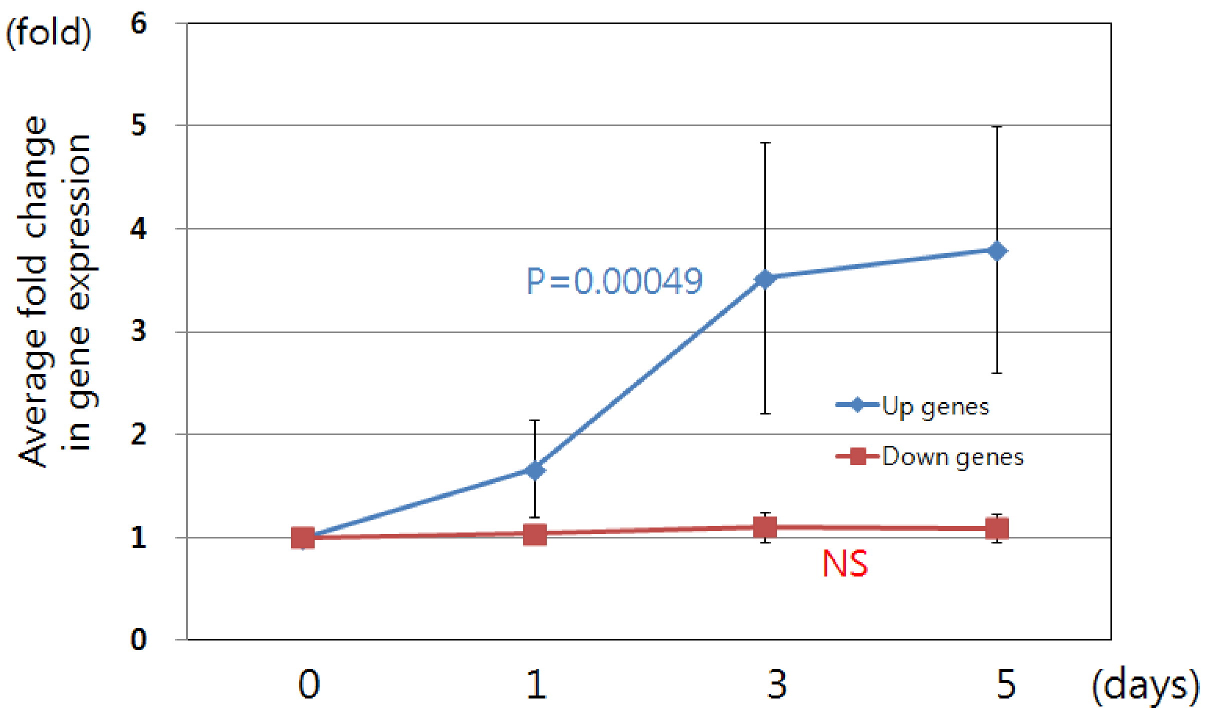

Figure S1-3. Gene expression of six litmus QC genes is increased in the Litmus cells exposed to plasma samples with different quality. Average fold changes in gene expression of 6 up-regulated genes (*IL-1B, PRG2*, *IL-8*, *CCL20*, *PTGS2*, *CEMID*) were increased during the time period of exposure of plasma samples to room temperature. P value indicates statistical significance of expression changes in six up-regulated genes at four different time points. In contrast down-regulated genes did not exhibit a significance. P value indicates a statistical significance of ANOVA test among different time points.

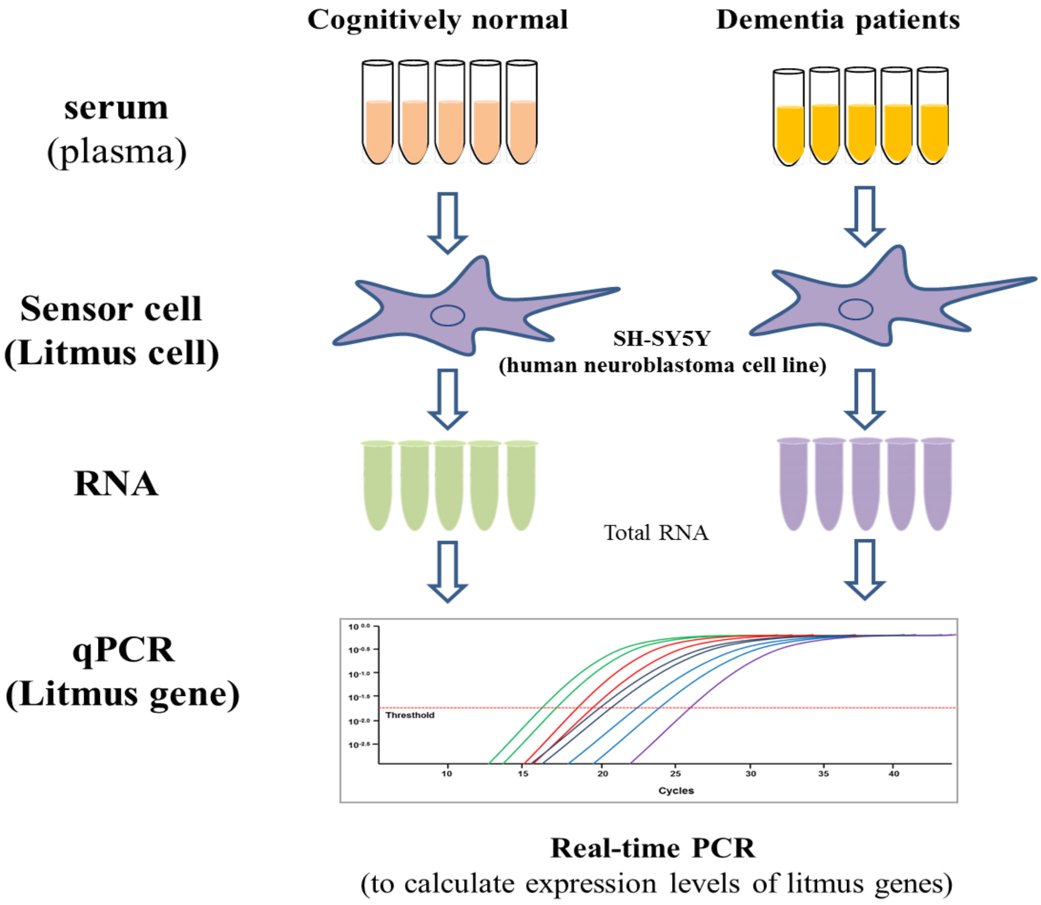

Figure S2. Screening strategy of dementia serum/plasma-responsive genes

Table S1-1. List of differentially expressed genes in decayed plasma-treated neuroblastoma cells

TargetID	RefSeq_NM	Gene	Fold change	Raw.p-value	Adj. p-value
ILMN_1775501	NM_000576.2	IL1B	25.42	8.81E-03	6.31.E-02
ILMN_1813704	NM_018689.1	KIAA1199	16.89	1.94E-02	1.09.E-01
ILMN_1729314	NM_002728.4	PRG2	10.89	3.72E-03	3.55.E-02
ILMN_2184373	NM_000584.2	IL8	9.73	6.83E-05	4.06.E-03
ILMN_1657234	NM_004591.1	CCL20	9.06	3.35E-04	8.50.E-03
ILMN_2054297	NM_000963.1	PTGS2	7.70	2.38E-03	2.68.E-02
ILMN_1771051	NM_000992.2	RPL29	6.56	2.48E-04	7.30.E-03
ILMN_1737517	NM_000992.2	RPL29	6.37	2.03E-04	6.57.E-03
ILMN_2064150	NM_000951.1	PRRG2	6.28	4.29E-06	1.83.E-03
ILMN_1666733	NM_000584.2	IL8	5.19	1.35E-05	2.22.E-03
ILMN_2357542	NM_003381.2	VIP	5.13	1.67E-03	2.14.E-02
ILMN_1792455	NM_015444.2	TMEM158	5.11	1.39E-03	1.91.E-02
ILMN_2188862	NM_004864.1	GDF15	4.74	6.97E-06	2.01.E-03
ILMN_1710954	NM_175901.3	LOC283932	4.50	5.04E-04	1.05.E-02
ILMN_1788107	NM_000641.2	IL11	4.29	1.52E-04	5.65.E-03
ILMN_1759453	NM_006294.2	UQCRB	4.21	1.40E-06	1.71.E-03
ILMN_1683456	NM_006273.2	CCL7	4.15	8.86E-03	6.33.E-02
ILMN_1683250	XM_933693.2	LOC440731	4.08	2.48E-03	2.75.E-02
ILMN_1794638	NM_194435.1	VIP	4.07	2.24E-03	2.58.E-02
ILMN_1697220	NM_002526.1	NT5E	3.92	3.29E-03	3.29.E-02
ILMN_1733847	NM_003857.2	GALR2	3.91	3.18E-05	2.95.E-03
ILMN_2399893	NM_001026.3	RPS24	3.77	1.02E-04	4.70.E-03
ILMN_1755664	NM_001029.3	RPS26	3.74	8.63E-07	1.71.E-03
ILMN_2339835	NM_080591.1	PTGS1	3.63	1.79E-03	2.24.E-02
ILMN_1677511	NM_000963.1	PTGS2	3.43	9.12E-04	1.47.E-02
ILMN_1661516	NM_001097615.1	POLR2J3	3.38	3.27E-06	1.74.E-03
ILMN_1795963	NM_013370.2	OKL38	3.36	2.40E-04	7.17.E-03
ILMN_1707727	NM_139314.1	ANGPTL4	3.23	9.40E-03	6.60.E-02
ILMN_1693826	NM_032782.3	HAVCR2	3.14	1.64E-05	2.34.E-03
ILMN_1694075	NM_001924.2	GADD45A	3.13	7.47E-04	1.31.E-02
ILMN_3280565	XM_945799.3	LOC389342	3.13	2.16E-02	1.17.E-01
ILMN_1752965	NM_013372.5	GREM1	3.11	2.49E-04	7.30.E-03
ILMN_1676984	NM_004083.4	DDIT3	3.08	3.97E-04	9.25.E-03
ILMN_1705750	NM_004613.2	TGM2	3.07	2.21E-02	1.19.E-01
ILMN_2052208	NM_001924.2	GADD45A	3.06	6.94E-04	1.26.E-02
ILMN_1693702	NM_203462.1	MRFAP1L1	3.00	1.88E-04	6.32.E-03
ILMN_1731891	NM_005671.2	UBXN8	2.96	1.24E-05	2.20.E-03
ILMN_1814106	NM_199001.1	C9orf169	2.88	1.06E-03	1.62.E-02
ILMN_2397028	NM_002640.3	SERPINB8	2.88	4.05E-06	1.81.E-03
ILMN_1714861	NM_001251.1	CD68	2.87	2.26E-03	2.60.E-02
ILMN_1743635	NM_144988.2	ALG14	2.85	1.31E-04	5.25.E-03
ILMN_1682717	NM_003897.3	IER3	2.85	2.22E-02	1.19.E-01
ILMN_1745860	NM_173677.1	FLJ40852	2.78	1.03E-06	1.71.E-03
ILMN_1666731	NM_015931.1	C3orf32	2.77	5.93E-04	1.15.E-02
ILMN_2334760	NM_177947.2	ARMCX3	2.76	1.06E-03	1.62.E-02
ILMN_2191822	NM_144988.2	ALG14	2.72	8.10E-05	4.30.E-03
ILMN_1789074	NM_005345.4	HSPA1A	2.69	2.14E-05	2.54.E-03
ILMN_1679417	NM_080599.1	UPF2	2.65	2.12E-05	2.54.E-03
ILMN_1789502	NM_001448.2	GPC4	2.64	5.25E-03	4.42.E-02
ILMN_2334765	NM_177947.2	ARMCX3	2.45	8.48E-04	1.41.E-02

TargetID	RefSeq_NM	Gene	Fold change	Raw.p-value	Adj. p-value
ILMN_2112811	NM_021029.4	RPL36A	2.44	1.72E-04	6.00.E-03
ILMN_2390310	NM_001001870.1	C17orf91	2.44	3.75E-04	9.01.E-03
ILMN_1689431	NM_153360.1	APCDD1L	2.42	1.69E-04	5.94.E-03
ILMN_1664861	NM_181353.1	ID1	2.39	1.50E-03	2.01.E-02
ILMN_1718960	NM_198833.1	SERPINB8	2.36	4.42E-05	3.39.E-03
ILMN_1698231	NM_015713.3	RRM2B	2.36	4.42E-04	9.79.E-03
ILMN_1770338	NM_014220.2	TM4SF1	2.35	8.88E-05	4.48.E-03
ILMN_1713124	NM_003739.4	AKR1C3	2.33	1.37E-04	5.37.E-03
ILMN_1750880	NM_207337.1	AMN1	2.32	1.82E-03	2.26.E-02
ILMN_3253126	XR_042107.1	FLJ41484	2.28	1.20E-02	7.77.E-02
ILMN_1784602	NM_000389.2	CDKN1A	2.28	6.36E-03	5.05.E-02
ILMN_1803429	NM_001001391.1	CD44	2.28	8.70E-03	6.25.E-02
ILMN_1806502	NM_003447.2	ZNF165	2.27	2.40E-06	1.71.E-03
ILMN_2170595	NM_015713.3	RRM2B	2.25	7.59E-04	1.33.E-02
ILMN_1743199	NM_000399.2	EGR2	2.24	2.20E-03	2.55.E-02
ILMN_1693014	NM_005194.2	CEBPB	2.23	6.14E-04	1.17.E-02
ILMN_1689004	NM_016639.1	TNFRSF12A	2.23	3.96E-05	3.28.E-03
ILMN_1740165	NM_017970.2	C14orf102	2.22	1.21E-04	5.08.E-03
ILMN_1705814	NM_182507.2	KRT80	2.22	2.31E-05	2.62.E-03
ILMN_3215954	XR_016115.2	LOC653079	2.21	1.05E-04	4.75.E-03
ILMN_1734611	NM_000710.2	BDKRB1	2.20	2.23E-04	6.93.E-03
ILMN_2090802	NM_032323.1	TMEM79	2.20	5.28E-05	3.60.E-03
ILMN_1740466	NM_017633.2	FAM46A	2.18	8.97E-05	4.50.E-03
ILMN_2071446	NM_015886.3	PI15	2.16	1.61E-05	2.32.E-03
ILMN_3248521	NM_005671.2	UBXN8	2.16	9.82E-06	2.14.E-03
ILMN_2151281	NM_031412.2	GABARAPL1	2.15	4.02E-04	9.29.E-03
ILMN_2374076	NM_199043.1	C14orf102	2.15	7.49E-05	4.15.E-03
ILMN_3244110	NM_001099684.1	FAM156B	2.14	2.54E-05	2.73.E-03
ILMN_2359907	NM_001251.2	CD68	2.14	6.34E-05	3.91.E-03
ILMN_1699574	NM_003873.4	NRP1	2.14	8.60E-03	6.20.E-02
ILMN_1753490	NM_194303.1	C10orf39	2.13	1.35E-05	2.22.E-03
ILMN_1658160	NM_014138.3	FAM156A	2.12	4.03E-05	3.30.E-03
ILMN_1787212	NM_078467.1	CDKN1A	2.11	2.42E-04	7.19.E-03
ILMN_3239236	NR_024348.1	NCRNA00095	2.11	3.04E-05	2.88.E-03
ILMN_1789702	NM_000158.2	GBE1	2.09	1.33E-05	2.22.E-03
ILMN_1758034	NM_004453.1	ETFDH	2.08	3.56E-03	3.46.E-02
ILMN_1720829	NM_003407.2	ZFP36	2.07	7.41E-04	1.31.E-02
ILMN_1768595	NM_001365.2	DLG4	2.06	2.34E-03	2.65.E-02
ILMN_1659106	NM_012396.3	PHLDA3	2.05	8.62E-03	6.21.E-02
ILMN_1873300	BI821208		2.05	3.61E-05	3.17.E-03
ILMN_2290808	NM_000982.3	RPL21	2.05	2.68E-04	7.56.E-03
ILMN_1695357	NM_017785.3	CCDC99	2.04	2.16E-06	1.71.E-03
ILMN_2151056	NM_144591.1	C10orf32	2.04	1.82E-03	2.26.E-02
ILMN_2188264	NM_001554.3	CYR61	2.03	7.43E-05	4.15.E-03
ILMN_2046730	NM_002966.2	S100A10	2.03	5.79E-03	4.74.E-02
ILMN_1700028	NM_016481.3	C9orf156	2.03	1.15E-03	1.70.E-02
ILMN_1717056	NM_001093771.1	TXNRD1	2.03	4.24E-04	9.56.E-03
ILMN_1670925	NM_144607.3	CYB5D1	2.02	8.98E-05	4.50.E-03
ILMN_1665554	NM_018310.2	BRF2	2.01	2.61E-04	7.45.E-03
ILMN_3237368	XR_017532.2	LOC644132	2.00	1.41E-06	1.71.E-03
ILMN_1778755	NM_014817.3	TRIL	-3.89	1.84E-06	1.71.E-03
ILMN_1801378	NM_017421.3	COQ3	-3.79	7.48E-04	1.31.E-02

TargetID	RefSeq_NM	Gene	Fold change	Raw.p-value	Adj. p-value
ILMN_1793724	NM_138807.2	C3orf31	-3.71	4.99E-07	1.43.E-03
ILMN_1690993	NM_024019.2	NEUROG2	-3.54	1.25E-03	1.79.E-02
ILMN_2077952	NM_020692.1	GALNTL1	-3.53	4.60E-03	4.08.E-02
ILMN_2380938	NM_004200.2	SYT7	-3.50	4.07E-05	3.30.E-03
ILMN_1772645	NM_018238.2	AGK	-3.41	1.89E-04	6.34.E-03
ILMN_1680618	NM_002467.3	MYC	-3.24	6.37E-04	1.20.E-02
ILMN_1737184	NM_031942.4	CDCA7	-3.22	1.44E-05	2.24.E-03
ILMN_1837428	AF131784		-3.11	1.88E-05	2.43.E-03
ILMN_1796069	NM_182511.2	CBLN2	-3.07	8.33E-06	2.14.E-03
ILMN_1773059	NM_032777.6	GPR124	-2.99	7.53E-06	2.02.E-03
ILMN_1679134	NM_017615.1	NSMCE4A	-2.94	2.95E-04	8.01.E-03
ILMN_1813295	NM_018640.3	LMO3	-2.92	4.92E-05	3.52.E-03
ILMN_1769694	NM_001095.2	ACCN2	-2.88	2.14E-05	2.54.E-03
ILMN_2399016	NM_001032278.1	MMP28	-2.87	5.66E-06	1.90.E-03
ILMN_1658486	NM_172251.1	MRPL54	-2.84	2.45E-06	1.71.E-03
ILMN_3250273	NM_014548.3	TMOD2	-2.84	1.77E-05	2.40.E-03
ILMN_1786658	NM_001035505.1	BOLA3	-2.83	2.07E-03	2.46.E-02
ILMN_2219767	NM_005378.4	MYCN	-2.81	5.71E-06	1.90.E-03
ILMN_1786065	NM_001048201.1	UHRF1	-2.78	6.47E-04	1.21.E-02
ILMN_1707084	NM_015983.2	UBE2D4	-2.71	7.23E-06	2.01.E-03
ILMN_1736242	NM_015432.2	PLEKHG4	-2.71	1.01E-03	1.58.E-02
ILMN_2077094	NM_013265.2	C11orf2	-2.69	1.18E-05	2.17.E-03
ILMN_1776076	NM_015352.1	POFUT1	-2.69	4.75E-05	3.49.E-03
ILMN_1798533	NM_006963.3	ZNF22	-2.68	1.62E-03	2.11.E-02
ILMN_1750181	NM_017899.2	TESC	-2.68	9.69E-04	1.53.E-02
ILMN_1654385	NM_024701.2	ASB13	-2.66	2.88E-05	2.83.E-03
ILMN_1687403	NM_003776.2	MRPL40	-2.66	6.75E-06	2.01.E-03
ILMN_2315780	NM_006997.2	TACC2	-2.65	4.43E-05	3.39.E-03
ILMN_2117904	NM_006963.3	ZNF22	-2.63	1.93E-03	2.34.E-02
ILMN_1681949	NM_006206.3	PDGFRA	-2.63	1.35E-05	2.22.E-03
ILMN_1777190	NM_001928.2	CFD	-2.61	1.20E-03	1.75.E-02
ILMN_2343010	NM_212552.2	BOLA3	-2.60	1.37E-03	1.89.E-02
ILMN_1665331	NM_000481.2	AMT	-2.60	3.05E-06	1.74.E-03
ILMN_1679949	NM_024103.2	SLC25A23	-2.58	5.38E-06	1.90.E-03
ILMN_3240370	NM_001085372.1	C11orf83	-2.58	1.16E-02	7.59.E-02
ILMN_1712298	NM_198401.2	ANKRD46	-2.54	1.57E-03	2.07.E-02
ILMN_3233179	XM_001132928.1	LOC728969	-2.53	2.38E-05	2.64.E-03
ILMN_1712913	NM_133369.2	UNC5A	-2.50	6.96E-05	4.08.E-03
ILMN_1795388	NM_032594.3	INSM2	-2.50	4.46E-05	3.39.E-03
ILMN_3241953	NM_033110.1	A2LD1	-2.48	7.27E-03	5.52.E-02
ILMN_1784985	NM_207351.3	PRRT3	-2.47	4.50E-04	9.89.E-03
ILMN_2086470	NM_006206.3	PDGFRA	-2.46	1.85E-07	1.40.E-03
ILMN_1718972	NM_138431.1	MFSD3	-2.44	8.22E-05	4.34.E-03
ILMN_3238435	NR_002954.1	SNORA12	-2.43	1.58E-04	5.77.E-03
ILMN_1814787	NM_004968.2	ICA1	-2.42	8.74E-06	2.14.E-03
ILMN_1804351	NM_003507.1	FZD7	-2.41	4.67E-04	1.01.E-02
ILMN_2384056	NM_001039966.1	GPER	-2.41	1.72E-07	1.40.E-03
ILMN_1691178	XM_939092.1	LOC650003	-2.40	3.67E-06	1.74.E-03
ILMN_1695020	NM_152720.1	NEK3	-2.40	7.17E-03	5.48.E-02
ILMN_2233099	NM_003146.2	SSRP1	-2.38	1.50E-04	5.60.E-03
ILMN_1701514	NM_147686.1	TRAF3IP2	-2.38	3.84E-04	9.13.E-03
ILMN_3251132	NM_001142885.1	TMOD2	-2.38	6.44E-05	3.96.E-03

TargetID	RefSeq_NM	Gene	Fold change	Raw.p-value	Adj. p-value
ILMN_1797172	NM_001983.2	ERCC1	-2.37	9.27E-03	6.54.E-02
ILMN_1701918	NM_001007256.1	KLHDC9	-2.37	2.06E-03	2.45.E-02
ILMN_2365569	NM_004968.2	ICA1	-2.37	2.11E-05	2.53.E-03
ILMN_1901198	BC071749		-2.37	6.91E-04	1.26.E-02
ILMN_2056815	NM_001004432.1	LINGO4	-2.37	8.21E-03	5.99.E-02
ILMN_2388800	NM_003713.3	PPAP2B	-2.35	1.18E-05	2.17.E-03
ILMN_1662340	NM_018083.4	ZNF358	-2.35	1.32E-05	2.22.E-03
ILMN_1795298	NM_001039966.1	GPER	-2.33	1.81E-05	2.41.E-03
ILMN_2304289	NM_021150.1	GRIP1	-2.32	6.95E-05	4.08.E-03
ILMN_1676555	NM_024926.1	TTC26	-2.32	7.85E-05	4.25.E-03
ILMN_1768812	NM_022003.1	FXYD6	-2.31	5.03E-04	1.05.E-02
ILMN_2110908	NM_002467.3	MYC	-2.30	3.08E-03	3.16.E-02
ILMN_1777233	NM_004091.2	E2F2	-2.29	2.70E-04	7.59.E-03
ILMN_1794829	NM_138409.1	C6orf117	-2.28	7.08E-04	1.27.E-02
ILMN_2328972	NM_006892.3	DNMT3B	-2.27	7.28E-04	1.30.E-02
ILMN_1693136	NM_006370.1	VTI1B	-2.26	4.71E-06	1.84.E-03
ILMN_1799015	NM_018663.1	PXMP2	-2.26	2.18E-03	2.55.E-02
ILMN_1735474	XM_939319.2	R3HCC1	-2.24	5.76E-07	1.43.E-03
ILMN_1779875	NM_006288.2	THY1	-2.24	1.28E-04	5.20.E-03
ILMN_2215545	NM_032359.2	C3orf26	-2.23	7.45E-06	2.01.E-03
ILMN_1687751	NM_001024372.1	BAALC	-2.23	1.01E-04	4.67.E-03
ILMN_3305304	NM_001127218.1	POLD2	-2.23	9.10E-05	4.51.E-03
ILMN_2225537	NM_012212.2	PTGR1	-2.22	5.10E-03	4.35.E-02
ILMN_1709124	NM_020959.1	ANO8	-2.22	2.78E-04	7.74.E-03
ILMN_1684306	NM_019554.2	S100A4	-2.20	9.79E-04	1.54.E-02
ILMN_2349459	NM_001168.2	BIRC5	-2.19	4.79E-05	3.49.E-03
ILMN_1754727	NM_001004051.1	GPRASP2	-2.18	8.40E-05	4.39.E-03
ILMN_1777740	NM_016647.2	C8orf55	-2.18	1.80E-05	2.41.E-03
ILMN_1810891	XM_034819.6	ZNF629	-2.18	1.04E-03	1.60.E-02
ILMN_2061565	NM_014638.2	PLCH2	-2.17	9.74E-05	4.64.E-03
ILMN_3272768	NR_023919.1	HSPC157	-2.16	9.07E-04	1.47.E-02
ILMN_3194087	NR_023918.1	HSPC157	-2.16	7.53E-04	1.32.E-02
ILMN_1791884	NR_003239.1	SNHG11	-2.16	1.53E-05	2.27.E-03
ILMN_1881909	BU536065		-2.16	9.56E-04	1.52.E-02
ILMN_2131392	NM_018034.2	WDR70	-2.16	2.35E-06	1.71.E-03
ILMN_2230566	NM_006822.1	RAB40B	-2.16	5.01E-06	1.88.E-03
ILMN_2139970	NM_000693.1	ALDH1A3	-2.15	4.04E-05	3.30.E-03
ILMN_1746465	NM_014344.2	FJX1	-2.15	7.00E-04	1.27.E-02
ILMN_1811921	NM_004078.1	CSRP1	-2.13	2.38E-02	1.25.E-01
ILMN_1786601	NM_002657.2	PLAGL2	-2.13	1.00E-04	4.66.E-03
ILMN_1704531	NM_012212.2	PTGR1	-2.13	2.06E-03	2.45.E-02
ILMN_1746116	NM_000275.1	OCA2	-2.13	8.81E-05	4.48.E-03
ILMN_1651557	NM_153705.4	KDELC2	-2.13	3.76E-04	9.02.E-03
ILMN_2345015	NM_198938.1	PTGES2	-2.13	3.30E-04	8.44.E-03
ILMN_1717599	NM_020872.1	CNTN3	-2.11	2.87E-05	2.83.E-03
ILMN_1772605	NM_006653.3	FRS3	-2.11	2.89E-03	3.03.E-02
ILMN_1790603	NM_022745.3	ATPAF1	-2.10	3.98E-07	1.43.E-03
ILMN_1753393	NM_017807.2	OSGEP	-2.10	8.08E-05	4.30.E-03
ILMN_1721921	NM_000386.2	BLMH	-2.10	1.98E-05	2.45.E-03
ILMN_2415583	NM_001077188.1	HS6ST2	-2.08	4.46E-05	3.39.E-03
ILMN_2093720	NM_017872.3	THG1L	-2.08	1.80E-03	2.25.E-02
ILMN_1706455	NM_138376.1	TTC5	-2.08	3.69E-05	3.20.E-03

TargetID	RefSeq_NM	Gene	Fold change	Raw.p-value	Adj. p-value
ILMN_1814797	XM_001132419.1	SLC35F3	-2.07	4.09E-05	3.30.E-03
ILMN_1772876	NM_018660.2	ZNF395	-2.07	3.66E-07	1.43.E-03
ILMN_2361096	NM_178151.1	DCX	-2.07	4.48E-04	9.86.E-03
ILMN_2081335	NM_018224.2	C7orf44	-2.07	1.91E-05	2.43.E-03
ILMN_1726589	NM_020404.2	CD248	-2.07	2.65E-04	7.52.E-03
ILMN_1698259	NM_018286.2	TMEM100	-2.07	1.01E-04	4.67.E-03
ILMN_1799969	NM_012437.3	SNAPIN	-2.07	4.34E-05	3.38.E-03
ILMN_1728106	NM_000594.2	TNF	-2.05	4.97E-02	2.05.E-01
ILMN_1807439	NM_000693.2	ALDH1A3	-2.05	4.34E-05	3.38.E-03
ILMN_1657893	NM_006440.3	TXNRD2	-2.05	3.05E-04	8.16.E-03
ILMN_1805028	NM_025075.1	THOC7	-2.05	1.01E-05	2.14.E-03
ILMN_3188110	NM_001100419.1	C19orf60	-2.04	2.66E-04	7.53.E-03
ILMN_1719158	NM_001012614.1	CTBP1	-2.04	1.72E-05	2.40.E-03
ILMN_1651544	NM_018486.1	HDAC8	-2.04	2.99E-04	8.06.E-03
ILMN_1695829	NM_032646.5	TTYH2	-2.04	1.09E-04	4.82.E-03
ILMN_1662328	NM_017623.4	CNNM3	-2.04	5.67E-05	3.70.E-03
ILMN_1779353	NM_019042.3	PUS7	-2.03	1.81E-04	6.19.E-03
ILMN_2082865	NM_015993.1	PLLP	-2.03	3.81E-04	9.09.E-03
ILMN_1781638	NM_198527.2	HDDC3	-2.03	1.93E-03	2.34.E-02
ILMN_1813834	NM_018137.1	PRMT6	-2.02	3.81E-05	3.24.E-03
ILMN_3235279	NM_018059.4	RADIL	-2.02	3.50E-05	3.13.E-03
ILMN_3251550	NM_007350.3	PHLDA1	-2.02	2.10E-03	2.48.E-02
ILMN_1685608	NM_002523.1	NPTX2	-2.02	2.19E-05	2.57.E-03
ILMN_2186858	NM_018335.2	C14orf131	-2.01	8.79E-05	4.48.E-03
ILMN_1702419	NM_001008783.1	SLC35D3	-2.01	2.26E-04	6.97.E-03
ILMN_1693410	XM_941876.1	BRI3BP	-2.00	5.25E-05	3.58.E-03
ILMN_2395285	NM_180703.1	U1SNRNPBP	-2.00	4.13E-06	1.83.E-03
ILMN_1730229	NM_032866.3	CGNL1	-2.00	2.22E-03	2.57.E-02
ILMN_1661895	NM_015886.3	PI15	2.00	6.03E-04	1.16.E-02

Table S1-2. Functional annotation clusters of DEGs affected in deuronal cells by RT-exposed of plasma samples

Annotation Cluster	Enrichment Score	Category	Term	Count	%	p-value	Benjamini
Cluster 1	3.14	GOTERM_BP_FAT	GO:0001568~blood vessel development	12	6.19	2.07E-05	0.03
		GOTERM_BP_FAT	GO:0001944~vasculature development	12	6.19	2.59E-05	0.02
		GOTERM_BP_FAT	GO:0001525~angiogenesis	9	4.64	8.03E-05	0.04
		GOTERM_BP_FAT	GO:0048514~blood vessel morphogenesis	10	5.15	1.77E-04	-
		GOTERM_BP_FAT	GO:0016477~cell migration	10	5.15	1.24E-03	-
		GOTERM_BP_FAT	GO:0006928~cell motion	13	6.70	1.81E-03	-
		GOTERM_BP_FAT	GO:0048870~cell motility	10	5.15	2.56E-03	-
		GOTERM_BP_FAT	GO:0051674~localization of cell	10	5.15	2.56E-03	-
Cluster 2	2.52	GOTERM_BP_FAT	GO:0043065~positive regulation of apoptosis	14	7.22	2.16E-04	-
		GOTERM_BP_FAT	GO:0043068~positive regulation of programmed cell death	14	7.22	2.31E-04	-
		GOTERM_BP_FAT	GO:0010942~positive regulation of cell death	14	7.22	2.42E-04	0.05
		GOTERM_BP_FAT	GO:0042981~regulation of apoptosis	19	9.79	5.55E-04	-
		GOTERM_BP_FAT	GO:0043067~regulation of programmed cell death	19	9.79	6.24E-04	-
		GOTERM_BP_FAT	GO:0010941~regulation of cell death	19	9.79	6.51E-04	-
		GOTERM_BP_FAT	GO:0043066~negative regulation of apoptosis	11	5.67	1.95E-03	-
		GOTERM_BP_FAT	GO:0043069~negative regulation of programmed cell death	11	5.67	2.17E-03	-
		GOTERM_BP_FAT	GO:0060548~negative regulation of cell death	11	5.67	2.21E-03	-
		GOTERM_BP_FAT	GO:0006916~anti-apoptosis	8	4.12	3.43E-03	-
		GOTERM_BP_FAT	GO:0006917~induction of apoptosis	9	4.64	1.11E-02	-
		GOTERM_BP_FAT	GO:0012502~induction of programmed cell death	9	4.64	1.13E-02	-
		GOTERM_BP_FAT	GO:0051726~regulation of cell cycle	8	4.12	3.78E-02	-
Cluster 3	1.73	GOTERM_BP_FAT	GO:0012501~programmed cell death	14	7.22	5.18E-03	-
		GOTERM_BP_FAT	GO:0008219~cell death	15	7.73	8.04E-03	-
		GOTERM_BP_FAT	GO:0016265~death	15	7.73	8.52E-03	-
		GOTERM_BP_FAT	GO:0006915~apoptosis	13	6.70	1.17E-02	-
Cluster 4	1.69	GOTERM_BP_FAT	GO:0006692~prostanoid metabolic process	4	2.06	7.18E-04	-
		GOTERM_BP_FAT	GO:0006693~prostaglandin metabolic process	4	2.06	7.18E-04	-
		GOTERM_BP_FAT	GO:0006690~icosanoid metabolic process	5	2.58	9.96E-04	-
		GOTERM_BP_FAT	GO:0033559~unsaturated fatty acid metabolic process	5	2.58	1.35E-03	-
		SP_PIR_KEYWORDS	prostaglandin biosynthesis	3	1.55	1.95E-03	-
		GOTERM_BP_FAT	GO:0001516~prostaglandin biosynthetic process	3	1.55	4.62E-03	-
		GOTERM_BP_FAT	GO:0046457~prostanoid biosynthetic process	3	1.55	4.62E-03	-
		KEGG_PATHWAY	hsa00590:Arachidonic acid metabolism	4	2.06	2.78E-02	-
		GOTERM_BP_FAT	GO:0046456~icosanoid biosynthetic process	3	1.55	3.45E-02	-
		GOTERM_BP_FAT	GO:0006631~fatty acid metabolic process	6	3.09	3.99E-02	-
		GOTERM_BP_FAT	GO:0006636~unsaturated fatty acid biosynthetic process	3	1.55	4.09E-02	-
Cluster 5	1.58	GOTERM_BP_FAT	GO:0048754~branching morphogenesis of a tube	5	2.58	3.32E-03	-
		GOTERM_BP_FAT	GO:0001763~morphogenesis of a branching structure	5	2.58	5.28E-03	-
		GOTERM_BP_FAT	GO:0001558~regulation of cell growth	7	3.61	1.03E-02	-
		GOTERM_BP_FAT	GO:0035239~tube morphogenesis	5	2.58	3.24E-02	-
Cluster 6	1.54	GOTERM_BP_FAT	GO:0006974~response to DNA damage stimulus	11	5.67	2.86E-03	-
		SP_PIR_KEYWORDS	dna repair	7	3.61	1.16E-02	-
		SP_PIR_KEYWORDS	DNA damage	7	3.61	1.60E-02	-
		GOTERM_BP_FAT	GO:0006281~DNA repair	8	4.12	1.83E-02	-
		GOTERM_BP_FAT	GO:0033554~cellular response to stress	12	6.19	1.84E-02	-
		GOTERM_BP_FAT	GO:0006259~DNA metabolic process	11	5.67	2.18E-02	-

The functional clusters of DEGs (based on the cutoff criteria │FC│ ≥ 2 and p-value < 0.05) were analyzed using DAVID database. The representative groups with an enrichment score of 1.5 above are presented.

Table S1-3. List of primer sequences for differentially expressed genes

Genes	Forward primer sequences	Reverse primer sequences
TRIL	5'-CTATCCGGGGACATCTTCGC-3'	5'-ACTGCACGAAGACAGTGAGG-3'
COQ3	5'-GGAAAAGAGGTGGGATCGTTTG-3'	5'-GTCCCACTGAGCTGGTTCTT-3'
TAM41	5'-CAAGGCTGGCTGGAGATAGA-3'	5'-CTTCTTCAGGCCTAGTCGCA-3'
NEUROG2	5'-CCAACTAAGATGTTCGTCAAATCCG-3'	5'-CTTCTTCGTCGGCGCTGGAT-3'
GALNTL1	5'-CACACACATGACGGAGGGAT-3'	5'-GCGGCTGTGAAAAGGAACTG-3'
SYT7	5'-TCAGCCTTAGCGTCACTGTC-3'	5'-CAGGCAACTTGATAGCCTTCTTC-3'
IL1B	5'-TCTGGGATTCTCTTCAGCCA-3'	5'-CCTGGAAGGAGCACTTCATCT-3'
PRG2	5'-AGTCTTCAGACGTTTAGTCAAGC-3'	5'-AGACTTGACCCTGGTTGAGC-3'
IL8	5'-CAGAGACAGCAGAGCACACA-3'	5'-GGCAAAACTGCACCTTCACA-3'
CCL20	5'-GCGAATCAGAAGCAGCAAGCA-3'	5'-GATGTCACAGCCTTCATTGGC-3'
PTGS2	5'-GATCCCCAGGGCTCAAACAT-3'	5'-TTAAGTCCACCCCATGGCCC-3'
CEMIP	5'-GATGTGCTCGTACTCCCTCG-3'	5'-CGGTTGTTGAGGCAATGTGG-3'
GAPDH	5'-CAGGGCTGCTTTTAACTCTGGTAA-3'	5'-GTGGAATCATATTGGAACATGTAAACC-3'

Table S2-1. Demographic characteristics of the discovery and validation sample sets used in the AD Litmus gene assay.

	Discovery set			Validation set					
	**NC**	**AD**	***p-value*** [a]	**NC**	**aMCI**	***p-value*** [a]	**NC**	**AD**	***p-value*** [a]
Individuals, n (F/M, sex)	5 (5/0)	5 (5/0)	-	28 (20/8)	28 (20/8)	-	33 (25/8)	33 (25/8)	-
Age, years	77.8 ± 2.2	79.2 ± 2.2	-	74.3 ± 6.3	74.2 ± 6.3	-	74.8 ± 5.9	75.1 ± 6.2	-
Education, years	8.6 ± 5.0	2.2 ± 2.1	0.04	6.3 ± 5.1	4.3 ± 4.4	-	6.7 ± 5.1	3.6 ± 4.5	0.01
MMSE	28.4 ± 2.5	15.4 ± 2.4	<0.001	26.8 ± 2.7	23.4 ± 3.6	<0.001	27.0 ± 2.7	15.3 ± 5.4	<0.001
CDR	0.0 ± 0.0	0.9 ± 0.2	0.001	0.1± 0.2	0.3 ± 0.3	0.001	0.1± 0.2	1.1 ± 0.8	<0.001
ApoE4 (%)	40	100		11.1	30.8		15.2	48.5	

Data are shown as mean ± standard deviation (SD).

[a]Significant differences between groups were evaluated by independent t-test.

Table S2-2. Selected genes up or down-regulated in serum-treated cells

TargetID	RefSeq_NM	Gene	Fold change	Raw.p-value
ILMN_1664802	NM_134265.2	WSB1	1.24	2.41E-03
ILMN_1681899	NM_032158.3	NSUN5C	-1.20	1.07E-02
ILMN_1727087	NM_000165.3	GJA1	1.21	4.13E-02
ILMN_1751816	NM_014060.1	MCTS1	1.21	2.35E-02
ILMN_1756079	NM_001024936.1	WASF1	1.20	4.42E-03
ILMN_1759954	NM_001099285.1	PTMA	1.20	1.69E-03
ILMN_1779663	NM_024917.4	TRMT2B	-1.24	4.29E-02
ILMN_1795474	XR_018325.1	LOC644131	1.20	1.40E-03
**ILMN_1814281**	**NM_020675.3**	**SPC25**	**1.22**	**3.24E-02**
**ILMN_1814924**	**NM_145037.1**	**FAM55C**	**-1.25**	**1.97E-02**
ILMN_1876838	AI948563		-1.21	1.35E-04
ILMN_1893375	BX400393		-1.21	3.44E-02
ILMN_2051373	NM_002497.2	NEK2	1.22	1.85E-02
ILMN_2059452	NM_001046.2	SLC12A2	1.21	3.00E-02
ILMN_2118910	NM_016058.1	TPRKB	1.25	1.17E-02
ILMN_2189869	NM_015962.4	FCF1	-1.27	4.69E-02
ILMN_2189870	NM_015962.4	FCF1	-1.32	3.12E-02
ILMN_2219712	NM_002129.2	HMGB2	1.20	9.67E-04
ILMN_2396697	NM_021956.2	GRIK2	1.23	2.75E-02
ILMN_3208715	XR_018394.2	LOC440063	1.20	1.03E-02
ILMN_3227315	XR_042330.1	LOC729009	1.24	4.34E-04
ILMN_3252359	XM_001725676.1	LOC100129191	1.20	3.85E-02

Table S2-3. List of primer sequences for differentially expressed genes

Species	Genes	Primer sequences (5'->3')
Human	GAPDH	forward: CAGGGCTGCTTTTAACTCTGGTAA
		reverse: GTGGAATCATATTGGAACATGTAAACC
	FAM55C	forward: CCCCCGGAGAGCGAAG
		reverse: CGTCTAACTTTCCTTCCAGCA
	SPC25	forward: GCAGAGAGGTTGAAAAGGCTG
		reverse: GGGCACTATCTGACACTTCAT
Mouse	Gapdh	forward: GCAGTGGCAAAGTGGAGATTG
		reverse: AGATGGTGATGGGCTTCCCG
	Spc25	forward: GAGTCGGAAGAGCTGACTGC
		reverse: CGCTGATTTCTGCAGTCCTT
